# Metacognitive Performance, the Tip-of-Tongue Experience, Is Not Disrupted in Parkinsonian Patients

**DOI:** 10.1155/2012/174079

**Published:** 2012-04-22

**Authors:** Justin D. Oh-Lee, Sarah M. Szymkowicz, Stefanie L. Smith, Hajime Otani

**Affiliations:** ^1^Department of Psychology, Central Michigan University, Health Professions Building, Room 2181, 1280 E. Campus Drive, Mount Pleasant, MI 48859, USA; ^2^Department of Psychiatry & Psychology, Behavioral Health Services, Lutheran Hospital, 1730 W. 25th Street, Cleveland, OH 44113, USA; ^3^Department of Psychology, Central Michigan University, Mount Pleasant, MI 48859, USA; ^4^Department of Psychology, DePaul University, Chicago, IL 60614, USA; ^5^Department of Psychology, Central Michigan University, 101 Sloan Hall, Mount Pleasant, MI 48859, USA

## Abstract

The present study investigated whether a form of metamemory, the tip-of-tongue phenomenon (TOT), was affected in patients with Parkinson's disease (PD). The PD patient (*n* = 22), age-matched elderly control (*n* = 22), and college student control (*n* = 46) groups were compared on a motor timing task and TOT measures. Motor timing was assessed using a cued hand-clapping task, whereas TOT was assessed using general knowledge questions. The results indicated that motor timing was significantly impaired in the PD group relative to both control groups. However, all of the TOT metacognitive measures: frequency, strength, and accuracy were statistically equivalent between the PD patients and elderly control groups, both of whom showed significantly better memory performance than college controls. These findings demonstrate that TOT metamemory is not compromised in PD patients, and that further insight into TOT mechanisms in PD may prove helpful in developing novel intervention strategies to enhance memory and general cognitive functions in these patients.

## 1. Introduction

Parkinson's disease (PD) is a chronic, progressive neurodegenerative disorder characterized by the selective degeneration of dopamine-producing neurons extending from the substantia nigra pars compacta (SNc) to the striatum [1]. Whereas motor symptoms are perhaps the most obvious and well-known clinical features of PD [2], there is also a constellation of nonmotor symptoms that precede motor symptoms, which are both debilitating and problematic [3]. Cognitive deficits are perhaps the broadest group of nonmotor symptoms, and these symptoms can be generally grouped into categories of impairments in visuospatial function [4], learning and memory [5], and executive function [6, 7]. These early cognitive symptoms are thought to be, at least partly, the result of a denervation of dopaminergic neurotransmission from the SNc [8] and may share common underlying pathogenic factors to motor symptoms manifested at later stages of the disease [9].

Dopamine dysfunction in PD has also been associated with a range of memory difficulties [10], including working memory [11–13], prospective memory [14, 15], emotional memory [16], and category memory [17]. Moreover, memory deficits are often observed before any clinically significant motor symptoms are detected [18], giving them both diagnostic and therapeutic relevance [19]. Unfortunately, the treatment of memory impairments is often difficult, particularly when multiple symptoms are present [3]. One potentially useful strategy for improving memory in PD is to promote one's ability to monitor and control one's own cognitive functions, also known as metacognition [20–22]. The rationale behind these investigations is that poor memory performance in PD may stem from poor monitoring and control of one's memory functions [23, 24]. Monitoring and control can occur at every stage of memory operation (i.e., encoding, storage, and retrieval), and the assumption is that one's awareness of the current state of memory at each stage or metacognitive knowledge is causally related to the control processes one would adopt (e.g., spending more time searching for the answer). Among PD patients, many of the studies have focused on metamemory processes referred to as feelings-of-knowing (FOK) judgments [25–27]. In the present investigation, another, albeit similar, type of metacognitive judgments, referred to as a tip-of-the-tongue (TOT) metamemory judgments, was investigated because there is some evidence to indicate that these two types of judgments are based on different processes, which may be supported by distinct neural basis. In the present study, we focused on TOT during retrieval, because for elderly population including PD patients, TOT judgments associated with retrieving the knowledge they already have are not only central in decision making and in day-to-day activities, but also has an important clinical and prognostic implications.

FOK judgments are one's judgment that a piece of information that cannot currently be recalled is still in memory and can be recognized correctly if shown it at a later point in time [28–30]. Several studies demonstrated that the integrity of the frontal lobe function associated with executive cognitive function appears to be closely related to FOK [28, 29, 31]. Patients with frontal lobe injuries have also shown deficits in FOK accuracy [32]. Further support for the link between FOK and PFC comes from studies which have demonstrated that FOK metacognitive abilities are severely impaired in PD patients [25–27], who exhibit PFC dysfunction that is associated with degeneration of the basal ganglia motor circuitry [33, 34]. Also consistent are results from prospective memory studies indicating that patients with PD were preferentially impaired on event-based or time-based prospective memory tasks with higher levels of executive control, possibly associated with prefrontal lobe dysfunction in PD [14, 15, 35].

The first behavioral investigation of FOK was conducted by Hart [30] using the RJR (recall-judgment-recognition) paradigm. In this paradigm, participants are asked to answer general knowledge questions. If they are unable to retrieve the answer, they are asked to judge whether they have the answer (even though they are unable to retrieve it at the moment) and would be able to recognize the answer if it is presented among distractors at a later point in time. The RJR paradigm has been used to assess FOK in both episodic (e.g., paired-associate lists) and semantic memory (e.g., general knowledge questions) [31, 36, 37]. In the present study, general knowledge questions were used because answering these questions represents semantic modality that involves judgments about already existing information that is well integrated in the semantic network [38–40].

A TOT experience reflects a state of mind in which people are temporarily unable to think of target words or sought after information but feel that retrieval is imminent. A TOT is a frustrating emotional experience of not being able to retrieve the information on demand, but having an intense feeling that the sought-after information will pop into one's mind at any moment [41–43]. This intense feeling of imminence that the sought-after information will come to consciousness [36] is what differentiates TOTs from FOKs [44]. It has been suggested that TOTs are qualitatively different from FOK experiences [31, 43, 45] and that TOT and FOK are disparate neuropsychological states, perhaps regulated by different neural substrates [29, 46].

Studies in the area of neuroimaging have indicated that brain activity differs during TOT and FOK experiences. TOT appears to be localized within specific frontal lobe areas, but FOK activity is more generalized throughout the frontal and limbic areas. Activity in the anterior cingulate cortex (ACC), right dorsolateral prefrontal cortex, and right inferior cortex is uniquely associated with TOT judgments and resolution during the retrieval process [46–48]. On the other hand, the same ACC-prefrontal regions together with left prefrontal regions along the inferior frontal gyrus and several parietal regions were associated with FOK judgments [46, 47]. These differences in the neural correlates are reflected, in part, by the overall selective effect of PFC functioning on FOK, but not on TOT experiences. This research together with multiple independent observations [31, 45, 48–50] indicates that different areas of the brain may be activated during TOT as opposed to FOK and further lends support for the notion that TOT and FOK are disparate cognitive states, perhaps regulated by different neural substrates. It is, therefore, possible that TOT, but not FOK, is localized within specific areas including the right prefrontal and ACC regions, and the damages to within these brain regions summate to produce impairments on tasks sensitive to TOT metacognitive processes. On the other hand, if these brain regions remain intact, TOT should not be impaired in these individuals. Based on these studies and current findings pointing to the dissociation between FOK and TOT judgments, with FOK, but not TOT, being closely linked to generalized PFC function, it could be predicted that TOT metacognitive function, subserved by localized and specific frontal areas, may remain unaltered in PD patients [29, 31, 46].

In regards to TOTs in PD, one study revealed that PD patients experience TOT-like states when given a variety of verbal and naming tasks, such that participants experienced word-finding difficulties in the absence of memory loss [51]. In this study, PD patients showed impaired semantic performances during confrontation naming and category naming tasks, suggesting a problem in cognitive strategies necessary for appropriate word production and naming [51]. Although these behavioral deficiencies share some characteristics of TOT phenomenon, TOT metamemory was not directly measured in their study. The present study utilizes aforementioned RJR paradigm with general knowledge questions to evaluate comprehensive measures of TOT metamemory: its frequency, strength, and accuracy, during semantic knowledge retrieval process.

The goal of this study was to examine whether there is a deficit in TOT in nondemented patients with PD. In the present study, groups of PD patients, elderly control participants, and college control participants were compared on a motor timing task and a TOT metamemory task. First, PD patients and control participants were compared on their ability to clap in time to a cued metronome beat to show that PD patients exhibit typical motor impairment [52]. Second, PD patients and control participants were compared on a TOT task based on general knowledge questions. Because motor timing impairment in PD is closely linked to dysfunction in PFC [52–56], and because TOT does not appear closely linked to PFC function, it was predicted that individuals with PD would not show compromised TOT metacognition. The absence of TOT deficit would provide evidence that cortical and subcortical networks mediating this function are most likely still intact in patients with PD. Having intact metamemory functions, such as TOT, is believed to be important for normal functioning of one's memory, such as selecting effective retrieval strategies [57], and consequently would lead to improvement in the quality of life for these patients [58, 59]. The present study was, therefore, designed to test the hypothesis that TOT metamemory is unimpaired in PD patients who demonstrate motor response timing deficits.

## 2. Method

### 2.1. Participants

Twenty two PD patients (13 men and 9 women) were recruited from various mid-Michigan Parkinson's disease support groups. These patients received $25 for their participation and were tested in their home or at a local community center. They ranged in age from 55 to 83 years (Table 1). PD diagnosis was made by the patient's primary neurologist prior to this study. Patients were asked to report the time elapsed since their first diagnosis of PD and asked to indicate their perceived symptom severity [60]. At the time of testing, the average disease duration of the PD patients was 8.52 years (SD = 5.80). Patients were assessed on their regular medication (levodopa = 14, dopamine agonists = 11, COMT inhibitor = 5, MAO-B inhibitor = 4, anticholinergic = 1, and NMDA receptor antagonist = 1, SSRI = 2, with the exception of one individual who was taking no medication). Twenty two normal elderly control participants (9 men and 13 women) were either spouses or family members of those PD patients. Elderly control participants received $15 for participation and were tested in their home or at a local community center. Elderly participants ranged in age from 49 to 86 years (Table 1). Forty six normal college control participants (23 men and 23 women) were recruited from the subject pool at Central Michigan University. They received extra course credit. Participants ranged in age from 18 to 34 years (Table 1). The guidelines for ethical treatment of human participants were followed with approval given by the IRB at Central Michigan University.

 Participant's age, educational background, and Mini-Mental State Examination (MMSE) scores are summarized in Table 1. The elderly control group did not differ from the PD with regard to age, education level, or MMSE scores.

### 2.2. Materials and Procedure

Participants, tested individually, were given verbal instructions as well as a written copy of the instructions. They were informed that they would be participating in a motor timing task, where they would be asked to clap in time to a cued metronome beat. The Groove metronome device and software were used (New York, NY) [61]. Participants were asked to put on a headphone and a touch-sensitive hand device. They then clapped their hands in time to a cued metronome beat, which provided an auditory feedback. Visual feedback was provided via the computer monitor, such that participants could see how many milliseconds they were off from the cued beat with each clap. Their average timing responses were recorded by the computer program in an average of milliseconds they were off from the cued sound. It also provided the percentage of times the participants responded early and late (% early/late) during each session. Timing scores ranged from 0 to 500 milliseconds, with zero indicating that the participant was clapping directly on time with the cued metronome beat. Participants performed three different sessions (1 minute, 2 minute, and 1 minute) with a short break (30–40 seconds) between each session. In addition, motor impairment was assessed by asking the PD patients the extent to which they felt physically impaired by PD, as well as to indicate the extent to which they felt mentally impaired by PD. Scores on this index range from 1 to 7, with 1 indicating no impairment and 7 indicating severe impairment.

TOT performance was assessed by 30 general knowledge questions from the Nelson and Narens [58] norms that were presented on a computer screen, one at a time. These questions were the same as those used by Widner et al. [31] and were selected from the questions that had a normative recall probability between .41 and .58. For each question, participants were asked to orally respond with (1) the answer they thought fit, (2) say “do not know” if they did not know the answer, or (3) indicate that they were having a TOT experience, where they believed that they knew the answer but they could not currently recall it and that the answer would pop into their mind at any moment. When participants indicated a TOT state, they were asked to provide the strength of TOT experience by indicating how strong this feeling was on a scale of 1–20 (1—extremely weak; 20—extremely strong). They were given 20 seconds for each question, and the experimenter wrote down the response given for each question. No feedback was provided. Next, participants were given a 4-alternative forced-choice recognition test. With each question, participants were asked to choose a correct answer without leaving any questions blank.

The accuracy of TOT responses was examined by a Goodman-Kruskal gamma correlation computed for each participant [31]. The gamma correlation measures the association between TOT reports and subsequent recognition performance. The assumption is that if one has the answer, but is unable to retrieve it for the moment, they are likely to be able to recognize it on a subsequent recognition test. Just like any other correlational measure, gamma correlation ranges from −1 to +1. The correlation of 0 indicates no association between TOT responses and recognition performance, whereas the correlation of +1 indicates the perfect association between TOT responses and recognition performance. It is difficult, if not impossible, to interpret the correlation of −1. The gamma is computed based on four measures created by a 2 × 2 matrix consisting of TOT (yes or no) and recognition (yes or no). The four measures are frequency counts of (a) the items with TOT responses that are correctly recognized, (b) the items with TOT responses that were not correctly recognized, (c) the items without TOT responses that were correctly recognized, and (d) the items without TOT that were not correctly recognized. The gamma score is computed by the following formula, gamma = (ad − bc)/(ad + bc). This means that when the sum of the two products (ad and bc) is zero, the gamma score is undetermined, and therefore, we had to exclude these participants from the analysis. Four participants from the PD patient group (18.2%), five participants from the elderly control group (22.7%), and three participants from the college student group (6.5%) were excluded. Because the percentage of excluded participants was not greater in the PD patient group than in the elderly control group, we proceeded with this analysis.

## 3. Results

### 3.1. Timing Performance

**Figure 1:**
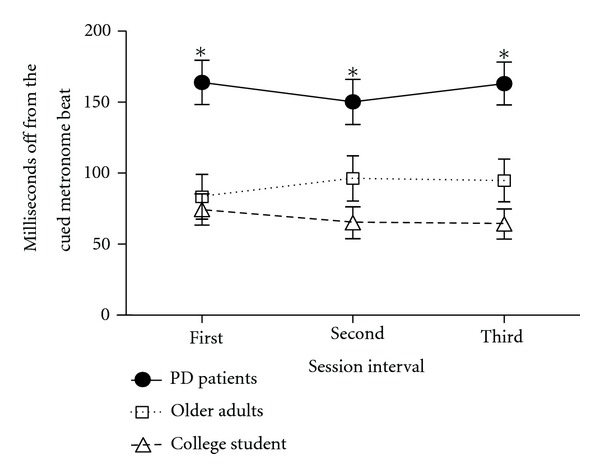
Motor timing performance (average of milliseconds off from the cued sound) for the first, second, and third sessions on the Groove for college students (open triangle), older control adults (open square), and Parkinson patients (filled circle). *P < .05 compared to college students.

Figure 1 shows the mean timing deviation from the cued metronome beat for the first, second, and third sessions for the PD patient, elderly control, and college student groups. As shown, the PD patients showed lower timing accuracy than the elderly control and college student groups. A 3 (group: PD patient, elderly control, and college student) × 3 (session intervals: first, second, and third) mixed-design analysis of variance (ANOVA) was conducted, with group as a between-subjects variable and session interval as a within-subjects variable. Due to a computer failure to record data, one college student was excluded from the analysis. The results indicated that the main effect of group was significant, *F*(2, 86) = 14.53, MSE = 12757.43, *P* = .0001, and *η*_p_^2^ = .25. However, the main effect of interval, *F*(2, 172) = 0.19, MSE = 1659.34, *P* = .83, and *η*_p_^2^^  ^ = .002, as well as the group × interval interaction, *F*(2, 172) = 1.00, MSE = 1659.34, *P* = .41, and *η*_p_^2^ = .02, was not significant. Fisher LSD tests comparing the three groups indicated that the PD patient group showed significantly lower timing accuracy (*M* = 159.06, SD = 81.84) than the elderly control (*M* = 91.47, SD = 59.38) and college student (*M* = 67.87, SD = 58.58) groups, with the latter two groups showing no difference from each other. It is also important to note that those PD patients who showed overall timing deficit had a greater proportion of their clapping responses occurring before the cued beat (indexed by % early responses) than the corresponding elderly control (≤70 years) and college student groups: *M* = 80%, 69%, and  76% early responses, respectively, *F*(1, 62) = 1.30, MSE = 255.7, and  *P* = .03. 

### 3.2. Memory Performance

**Figure 2:**
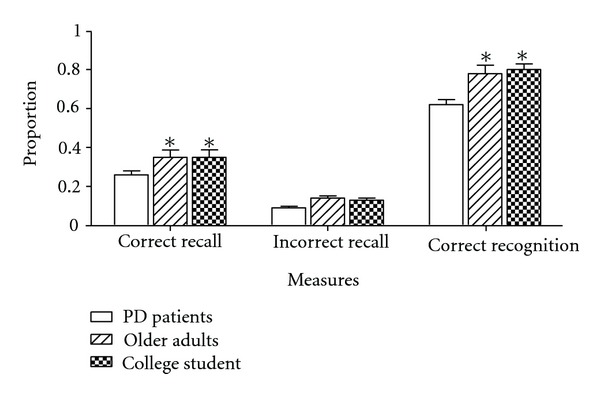
Recall and recognition performance expressed in proportion of the total number of general knowledge questions (30 questions) for college students (open bars), older adults (hatched bars), and Parkinson patients (stippled bars). *P < .05 compared to college students.

Figure 2 shows the mean proportion of (1) correctly recalled items, (2) incorrectly recalled items, and (3) correctly recognized items. Because the recognition test was a forced-choice test, the false alarm rate is the inverse of the mean correctly recognized items. As shown, all three measures showed comparable performance between PD patients and elderly control participants. These two groups, in turn, performed better than college students. A one-way between-subjects ANOVA was used to compare the three groups on each measure. For the correctly recalled items, the difference among the groups was significant, *F*(2, 87) = 3.13, MSE = 0.29, *P* = .049, and *η*_p_^2^ = .07. Fisher LSD tests showed that the difference between the PD patient (*M* = .35, SD = .18) and elderly control (*M* = .35, SD = .17) groups was not significant. Fisher LSD tests further showed that these two groups recalled a significantly greater number of correct answers than the college student group (*M* = .26, SD = .16). For the incorrectly recalled items (i.e., commission errors), the difference among the groups was significant, *F*(2, 87) = 7.86, MSE = 0.003, *P* = .001, and *η*_p_^2^ = .15. Fisher LSD tests showed that the PD patient (*M* = .13, SD = .50) and elderly control (*M* = .14, SD = .06) groups were not different from each other. The college student group (*M* = .09, SD = .06) showed significantly fewer incorrectly recalled items than the PD patient and elderly control groups. The correctly recognized items showed a similar pattern. The difference among the groups for correctly recognized items was significant,  *F*(2, 87) = 10.20, MSE = 0.03, *P* = .0001, and *η*_p_^2^ = .19. Fisher LSD tests showed that the difference was not significant between the PD patient (*M* = .80, SD = .15) and elderly control (*M* = .78, SD = .20) groups. These two groups, in turn, outperformed the college student group (*M* = .62, SD = .18). These results indicated that memory performance was similar between the PD patient and elderly control groups. Both the PD patient and elderly control groups showed better performance than the college student group. PD patient group showed no evidence of memory deficiency on general knowledge questions. 

### 3.3. Metamemory Performance

**Figure 3:**
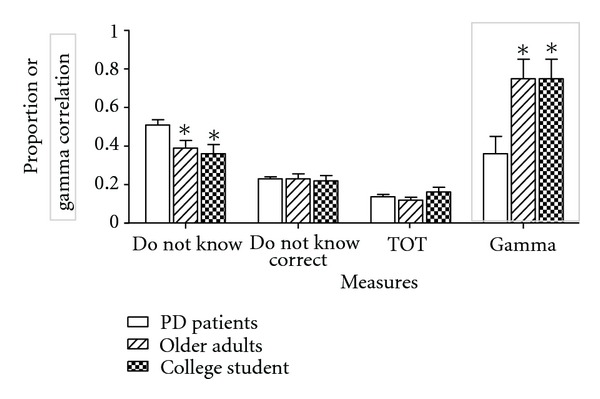
Do not know and TOT reports (in proportion to 30 general knowledge questions), TOT accuracy (gamma correlation), for college students (open bars), older adults (hatched bars), and Parkinson patients (stippled bars). *P < .05 compared to college students.

Figure 3 shows the mean proportion of (1) “do not know” responses, (2) correctly recognized items that participants said “do not know” (“do not know correct”), and (3) TOT responses across PD patients, elderly control participants, and college students. Figure 3 also shows TOT accuracy across the three groups, measured by Goodman-Kruskal gamma correlation between TOT responses and recognition performance [31, 62]. As shown, PD patients and elderly control participants showed similar performance on the “do not know” and “do not know correct” measures. For each measure, we conducted a one-way between-subjects ANOVA comparing the three groups. The results showed that the difference among the groups was significant for the “do not know” measure, *F*(2, 87) = 5.61, MSE = 0.04, *P* = .005, and *η*_p_^2^ = .11. Fisher LSD tests showed that the PD patient (*M* = .36, SD = .22) and elderly control groups (*M* = .39, SD = .20) made fewer “do not know” responses than the college student group (*M* = .51, SD = .18). No difference was found between the PD patient and elderly control groups. The results for the “do not know correct” measure revealed that the difference among the group was not significant, *F*(2, 87) = 0.03, MSE = 0.01, *P* = .97, and *η*_p_^2^ = .001, indicating that the accuracy of “do not know” responses was similar among PD patients (*M* = .22, SD = .12), elderly control participants (*M* = .23, SD = .12), and college students (*M* = .23, SD = .07). Next, we analyzed the total number of TOT responses. The results showed that the difference among the groups was not significant, *F*(2, 87) = 1.27, MSE = 0.01, *P* = .29, and *η*_p_^2^ = .03, indicating that the PD patient (*M* = .16, SD = .11), elderly control (*M* = .12, SD = .07), and college student (*M* = .13, SD = .09) groups showed similar TOT responses (expressed as a proportion of total number of responses). Because the PD and elderly control groups showed higher recall than the college student group, TOT responses were conditionalized on unrecalled items (i.e., TOT plus “do not know” responses); that is, what proportion of unrecalled items participants responded with TOT responses? One-tailed planned *t*-tests were conducted because the literature indicated that elderly adults tend to experience TOT at a higher frequency than young adults [37, 63]. The results indicated that the difference between the PD patient (*M* = .35, SD = .23) and college student groups (*M* = .22, SD = .13) was significant, *t*(66) = 2.99, *P* = .002. The difference between the elderly control (*M* = .28,SD = .24) and college student groups also approached significance,  *t*(66) = 1.41, *P* = .08. Because no difference was found between the PD patient and elderly control groups, *t*(42) = 0.97, *P* = .34, these two groups were combined and compared with the college student group. The difference was significant, *t*(88) = 2.44, *P* = .01, indicating that the older group (*M* = .32, SD = 24) showed a higher tendency of making TOT responses than the younger group (*M* = .22, SD = 1.3). 

In terms of TOT accuracy, the groups differed on gamma scores, *F*(2, 78) = 4.63, MSE = 0.19, *P* = .01, and *η*_p_^2^ = .11. Fisher LSD tests showed that the gamma scores were similar between PD patients (*M* = .72, SD = .44) and elderly control participants (*M* = .75, SD = .40). The gamma score was significantly lower for the college student group (*M* = .36, SD = .61) than the PD patient and elderly control groups, indicating that the accuracy of TOT responses was lower for the college student group than for the PD patient and elderly control groups. Recently, a question was raised as to whether gamma is the best measure of metacognitive accuracy. Benjamin and Diaz [64] recommend two alternative measures, signal detection (d_a_) and G*. Unfortunately, the former is not suitable for the present data because it requires at least a 2 × 3 contingency table. Furthermore, G* discards those participants who showed perfect accuracy. In fact, using G*, only six participants remained in the PD patient and elderly control groups because many participants in these groups were very conservative. Using the remaining participants, a one-way ANOVA did not show a difference among the groups, *F*(2, 37) = 0.15, MSE = 1.07, *P* = .86, and *η*_p_^2^ = .01. We also computed Hart difference score statistic (D) [65], which, according to Benjamin and Diaz, did better than gamma in their simulation. The results of a one-way ANOVA based on D also showed a nonsignificant difference among the groups, *F*(2, 79) = 0.12, MSE = 0.07, *P* = .89, and *η*_p_^2^ = .003. All these measures provided converging evidence that the PD patients did not show impairment in TOT accuracy.

To assess the effects of age and the amount of general knowledge on gamma correlation (i.e., TOT accuracy), an analysis of covariance (ANCOVA) was conducted using the age and the number of correctly recognized answers as variables. When the low, medium, and high knowledge groups were compared with age as a covariate, no difference was found among the groups, *F*(2, 74) = 1.21, MSE = 0.30, *P* = .30, and *η*_p_^2^ = .03. The difference was also nonsignificant when the young, middle, and old age groups were compared with general knowledge as a covariate, *F*(2, 31) = 1.40, MSE = 0.14, *P* = .26, and *η*_p_^2^ = .08. However, age as well as the number of correctly recognized answers significantly correlated with gamma score: age *r*(78) = .26, *P* = .02, and correctly recognized answer *r*(78) = .24, *P* = .04. Further, age was significantly correlated with the number of correctly recognized answers, *r*(90) = .40, *P* < .001. A linear regression showed that age and the number of correctly recognized answers jointly accounted for 9% of variance (*R* ^2^ = .09), *F*(2, 75) = 3.58, *P* = .03. However, neither alone significantly predicted TOT accuracy.

We also predicted that the MMSE score would be positively correlated with the TOT accuracy for the PD patients and elderly. The results showed that the correlation was nonsignificant for the PD patients, *r*(18) = .24, *P* = .17 (one-tailed) but significant for elderly, *r*(17) = .43, *P* = .04 (one-tailed). Combining the PD patients and elderly, the correlation was significant, *r*(35) = .32, *P* = .03 (one-tailed). In contrast, the correlation was much smaller for college students between the TOT gamma and the MMSE score, *r*(43) = .07, *P* = .33 (one-tailed). In summary, TOT accuracy and MMSE are related in PD patients and elderly, but not for college controls.

The strength of TOT states showed similar patterns. A mean TOT strength was computed for each participant using all TOT responses as well as using only TOT responses that were accurate (i.e., the ones that participants selected correct answers on the subsequent recognition test). Because both analyses produced similar results, the results from the former analysis will be presented here. An attempt was also made to compute the mean strength of inaccurate TOT; however, due to a small number of these responses, we were unable to proceed with this analysis. A one-way between-subjects ANOVA comparing all three groups showed that the difference was significant, *F*(2, 80) = 4.81, MSE = 12.52, *P* = .01, and *η*_p_^2^ = .11. Fisher LSD tests showed that the elderly control group (*M* = 13.37, SD = 4.66) showed higher strength than the college student group (*M* = 10.47, SD = 3.26). The PD group (*M* = 12.00, SD = 2.75) was not different from either group. In sum, there was no evidence that PD patients had metamemory deficiency. Both the PD patient and elderly control groups performed similarly on all metamemory measures: frequency, strength, and accuracy of TOT.

Based on the hypothesis testing procedures reported above, there was no statistical difference between the PD and elderly control groups. However, the failure to reject the null hypothesis does not mean that the two groups are equivalent because the *P* value is “the probability of data given that the null hypothesis is true” rather than “the probability that the null hypothesis is true given the data” [66, page 372]. To further support the equivalence of the two groups, the procedure that would establish statistical equivalence, described by Tryon [66] and Tryon and Lewis [67], was performed for each memory and metamemory measure as follows. First, the 95% inferential confidence interval (ICI) was computed for each group based on the revised formula described by Tryon and Lewis [67, Equation  9, page 274]. If there is an overlap in 95% ICI between the PD and elderly control groups, the difference is not statistically significant. Then, the question becomes whether the two groups are equivalent or the decision is indeterminant. Second, the value of the delta (Δ) was set. This is the amount of difference between the two groups “that is considered to be inconsequential” based on “substantive grounds that have been established apart from the analysis at hand by professional consensus or other means” [67, page 273]. There is no fast and easy method of determining Δ; however, for the purpose of the present investigation, we used the width of the 95% confidence internal (CI) for the elderly control group because if the PD group is equivalent to the elderly control group, the likely location of the population mean should be the same between these two groups. Third, the equivalence range (_*e*_ *R*_*g*_^2*α*^) was computed for each measure based on the procedure described by Tryon and Lewis. This range was based on 100 (1 − 2*α*)% ICI, using the shrinkage factor (*E* ^2*α*^). Equivalence between two groups is established when _*e*_ *R*_*g*_^2*α*^ ≤ Δ [67, Equation  23, page 276]. The assumption is that the difference between the lowest and the highest ends of 90% ICI (based on both groups) should not exceed Δ. As shown in Tables 2 and 3, for memory performance and metamemory performance, respectively, statistical equivalence is demonstrated in all but the TOT measure. However, when the TOT responses were conditionalized on unrecalled items (i.e., proportion of TOTs in unrecalled items), Δ (.19) did not exceed _*e*_ *R*_*g*_^2*α*^ (.21), indicating that the two means were equivalent. 

## 4. Discussion

 The present study investigated whether a form of metacognition, tip-of-tongue (TOT), was affected by Parkinson's disease (PD). Patients with PD showed lower motor response timing accuracy but uncompromised TOT performance, compared to both control groups. Both the PD patient and elderly control groups showed similar level on all TOT measures: frequency, strength, and accuracy, despite the fact that the PD patients showed significantly worse timing accuracy than the elderly control and college student groups. Further, general knowledge was uncompromised by PD on both recall and recognition tests; the PD patient group showed a similar level of performance to the elderly control group, which was higher than that of the college student control group.

 A variety of neuropsychological studies have reported a deficit in the ability of PD patients to accurately perceive duration and correctly timed motor responses [56, 68]. Accordingly, it was expected that PD patients would show lower timing accuracy relative to controls (elderly and college students) in performing the metronome timing task. The metronome timing task used in the present study involves several components of the working memory functions, including maintenance, manipulation, and monitoring of cognitive resource as related to timing interval, internal and external cues, and stimuli pacing [69, 70]. The present results agree with previous reports that PD patients have significantly worse motor timing than age-matched controls, suggesting that the brain regions affected in PD may play a direct role in such tasks [53]. In fact, a large number of studies have shown that human motor timing behavior is closely linked to the prefrontal cortex (PFC) and the basal ganglia activity, brain regions severely compromised in PD [54, 68, 71]. The present results are therefore consistent with the hypothesis that motor timing performance deficit in PD patients is closely related to a dysfunction of the prefrontal-basal ganglia circuits.

In contrast to the motor timing performance, there was no evidence that PD patients had deficiency in TOT metacognition. Both PD patients and elderly groups showed significantly greater TOT responses as well as TOT accuracy than did college group, showing about 200% and 206% accuracy increase compared with college group, respectively. In fact, both the PD patient and elderly control groups performed similarly on all metamemory measures: frequency, strength, and accuracy of TOT. It has been suggested that TOTs are simply strong FOK experiences [72], but evidence that refutes this claim has been reported. For example, Widner et al. [31] using perseveration errors made during the Wisconsin Card Sorting Task, as an index of PFC functioning, reported that the TOT judgments were not related to PFC functioning. In their study, PFC functioning had an impact on FOKs, but did not impact TOTs. The present results are compatible with a view that the TOT and FOK are disparate cognitive processes subserved by different neural substrates. The results also support a notion that the activation of the localized specific brain regions including the right prefrontal and ACC regions may be closely associated with the neuropsychological processes involved in both TOT judgment and retrieval. PD patients in the present study did not show impairments in TOT experience, and, therefore, it is conceivable that these brain regions remained intact and functional. In fact, the present results revealed that the MMSE scores were positively correlated with TOT accuracy scores in PD patients and elderly, further supporting the view that PD patients in the present study did not have damage in frontal lobe areas associated with TOT metacognition as well as with certain aspects of cognitive control.

In addition, although PD is associated with emotional impairment [16, 73, 74], a feeling of imminence and emotional reactions [75–77] that often accompanies TOT experience appears to be unaffected in PD patients. Taken together, the present findings support the postulate that TOT metacognitive function may not be directly related to dopamine deficiency in PD patients, which influence the overall prefrontal executive networks, but perhaps is likely due to dysfunction within a localized specific neural network [29, 46]. Future research, such as fMRI morphometry, will be needed to further delineate the precise nature of metacognitive and associated neural function in normal subjects as well as PD patients. Elucidating and characterizing a specific neural function associated with unimpaired TOT metacognition in PD patients may have significant therapeutic and prognostic implications for PD and other dopamine-related disorders.

 Uncompromised TOT metacognition among the PD patients may also depend on the nature of the memory task. In regard to another metacognitive measure FOK, some studies showed impaired FOK among PD patients [27, 78], whereas others showed intact FOK among PD patients [25, 26]. The disparate results are likely to be based on the difference in the nature of the memory task; that is, those studies that used an episodic memory task showed impaired FOK [27, 78], whereas those studies that used a semantic memory task showed spared FOK [26, 79]. Similarly, PD patients may show impaired TOT if the task is episodic in nature (e.g., paired-associate learning) rather than semantic in nature (e.g., general knowledge questions). In fact, in the present study, there was no evidence of impaired semantic memory by the PD patient group relative to both control groups; both correct recall and recognition showed comparable performance between PD patients and elderly participants, both of which, in turn, performed much better than college students. The episodic, but not semantic [38, 39], memory is thought to involve context retrieval and executive control operations that are closely linked to prefrontal cortical (PFC) executive functions [27, 32, 80, 81]. Given this, the present results appear to be in line with the hypothesis that PFC deficit in PD patients is closely related to a dysfunction of the episodic, but not semantic metacognition [27, 46, 82].

 One weakness of the present study was that metacognitive control was not directly measured. That is, TOT is an indicator of metacognitive knowledge; however, how this knowledge is used to control retrieval might show impairment among PD patients. A future question should be whether participants would show longer retrieval time when they experience TOT. This question could be answered by measuring participants' response time in reporting TOT experience as well as response time associated with TOT resolution.

 In regard to TOT and aging, it is well established that older adults experience more TOTs than younger adults, possibly due to age-related cognitive changes [37, 83]. The present results were consistent with these findings because when TOT responses were conditionalized on unrecalled items, older adults (PD patients and elderly control groups combined) showed a higher tendency of responding with TOT than the college student control group.

In terms of TOT accuracy, the present results were consistent with the incremental and metacognitive perspectives which argue for TOT accuracy increasing with aging as a result of knowledge culmination and memory network expansion [31, 84]. Conceivably, broader and stronger knowledge base in older adults including PD patients may have contributed to providing more familiar cues (the cue familiarity heuristics) and more target-related information (the accessibility heuristics) to increase the TOT accuracy [23, 26, 85]. This interpretation based on the increment view is consistent with the current data, in that the level of general knowledge (indexed by the number of correctly recognized answers), as well as age, covaried with TOT accuracy, suggesting that participants who had more knowledge were more likely to have more accurate TOT metacognitive judgments, regardless of age.

In regard to motor timing performance and the TOT metacognitive function, there was a dissociation between the accuracy of TOT judgments and the level of motor timing-related functioning. The results indicated that TOT accuracy (indexed by gamma correlations) was not affected by the level of motor timing accuracy (low- versus high-accuracy group) in any of the subject groups examined. There was also no correlation between motor timing accuracy and gamma correlation scores in any groups. The data indicated that TOT metamemory and motor timing performance may represent distinct cognitive functions, potentially mediated by functionally and structurally different systems from one another. To the extent that the motor timing abilities are related to the prefrontal cortical (PFC) function [68, 70], our results suggest the possibility that TOT experiences are indeed different from other metacognitive processes that require PFC function, such as in FOK and prospective memory performance [27, 31, 43]. Consistent with this theme, both FOK metamemory and prospective memory are impaired in PD [14, 15, 25, 26]. Further study examining both FOK and TOT in the same PD patient group, however, is required before any concrete conclusions can be established. Nevertheless, based on the current data, it can be concluded that despite patients with PD showing significant motor response timing impairment (an index of prefrontal and working memory impairment), they were not impaired in their TOT metamemory performance compared to age-matched adults.

One caveat to above interpretations is that PD patients examined in the current study were ON anti-Parkisonian medication, primarily dopaminergic agonists (DA). Despite inconsistent and incomplete reports on the effects of DA drugs on motor timing functions, [86], administration of DA agonist has been found to cause alterations in these functions including clock speed shifts to an earlier time, relative to the feedback time [52]. In the current study, PD patients displayed this effect with a greater proportion of clapping responses occurring early, relative to the cue (~16% greater shift to early responses), than did age-matched adults (80% and 69% early responses by PD and elderly, resp.). Consequently, it is difficult, if not impossible, to rule out the possibility that the null difference in TOT measures between the PD patient and elderly control groups might have resulted from an anti-Parkinsonian drug-related effect. More accurate and careful interpretation of current data must await further research that examines the effects of DA drugs on metacognition and motor timing learning in PD patients while ON or OFF medication.

In view of the previously reported association between metacognitive abilities with competence in problem solving and learning new skills [20, 87, 88], the present results appear to be relevant to efficiency in memory retrieval process and quality of life for PD patients [15, 43, 89]. Conceivably, PD patients without dementia could benefit from intervention strategies (e.g., implementation intention) that are based on metacognitive abilities (e.g., metacognitive knowledge, skill, and belief) in ways that will help promote qualities such as self-evaluation, self-monitoring, self-control, self-motivation, and everyday functional capability [88, 90–92]. Being in a TOT state is often frustrating experience, but it may be used as a cue or strategy (think some more immediately, think some more later, or look up the answer) to facilitate retrieval [57]. Accordingly, PD patients may be trained to use TOT states to select effective and appropriate retrieval strategies in ways that will help improve their overall cognitive functions.

The future investigation should also include metacognitive judgments that are made at encoding or judgments of learning (JOL), which is one's judgment that a given item is learned adequately enough for successful retrieval on a future test. Research has shown that JOLs are causally related to monitoring and control of the acquisition of to-be-learned materials [49, 93–95]. The issue is whether PD patients would be able to use JOL for effective monitoring and control of their operations during acquisition. Previous studies have shown that frontal lobe damages would be likely to lead to impairment in JOL [96] even though no JOL impairment was found when medial prefrontal cortex was damaged [97]. Taken together, it could be predicted that JOL metacognitive function may also remain uncompromised in PD patients. If this is the case, it will confer unique opportunities for development of safer and more effective cognitive intervention strategies in patients with PD as well as other related neurodegenerative diseases. Given the close link between cognitively demanding tasks on postural and motor stability in PD patients [98, 99], further study into this issue is obviously critical to better understand the contribution of metacognitive abilities in the human memory retrieval process, as well as in the overall cognitive and motor functions. The present findings also provide further support to the existing literature on cognitive abilities, which are not as severely impaired as was once purported in PD, including selective attention, decision-making, verbal memory, and adaptive abilities [26, 100–103]. By fully understanding which cognitive neural functions are unimpaired in PD, more effective training strategies can be developed to improve patient's symptom management strategies and their quality of life.

## 5. Conclusions

 The present findings, collectively, support the view that TOT metamemory judgment is not impaired in PD patients, and that varied metacognitive functions (TOT, FOK, and prospective memory) may be differentially affected by the disease. The findings also suggest potential implications of TOT metacognitive abilities related to memory retrieval processes as well as to general cognitive and motor functions in PD. Further study into this issue is obviously critical to the development of novel behavioral therapeutic options that may prove useful in enhancing memory function and overall quality of life in these patients.

## Figures and Tables

**Table 1:** Characteristics of participants (means and standard deviations).

	College control (*n* = 46)	Elderly control (*n* = 22)	PD (*n* = 22)
Age	20.65 (3.54)	68.41(9.70)*	71.50 (8.04)*
Education (in years)	13.26 (1.60)	13.36 (2.11)	14.36 (3.13)
MMSE	27.87 (2.00)	26.55 (2.37)*	25.36 (2.80)*
Age at diagnosis (PD)	—	—	62.91 (10.47)
Duration of disease	—	—	8.52 (5.80)

*P < .05 compared to college students. PD = patients with Parkinson's disease.

**Table 2:** Shrinkage factors (*E* ^*α*^ and *E* ^2*α*^), confidence intervals (CI), inferential confidence intervals (ICI), _*e*_ *R*_*g*_^2*α*^, and Δ for memory performance.

	Correct recall		Incorrect recall		Correct recognition	
	PD	Elderly	PD	Elderly	PD	Elderly
*M*	.35	.35	.13	.14	.80	.78
SD	.18	.17	.05	.06	.15	.20
SE	.04	.04	.01	.01	.03	.04
*E* ^*α*^	.69		.69		.69	
*E^2^* ^*α*^	.69		.69		.70	
95% CI	.27–.43	.28–.43	.11–.15	.11–.16	.73–.87	.69–.87
95% ICI	.29–.40	.30–.40	.11–.14	.12–.15	.75–.85	.72–.84
90% ICI	.30–.39	.31–.45	.11–.14	.12–.15	.76–.84	.73–.83
_*e*_ *R*_*g*_^2*α*^	.09		.04		.11	
Δ	.15		.04		.18	

Note: *N* = 22; *E* ^*α*^ is based on 100  (1 − *α*) and *E* ^2*α*^ is based on (1 − 2*α*); _*e*_ *R*_*g*_^2*α*^ is based on 90% ICI and Δ is based on 95% CI for the elderly group.

**Table 3:** Shrinkage factors (*E* ^*α*^ and *E* ^2*α*^), confidence intervals (CI), inferential confidence intervals (ICI), _*e*_ *R*_*g*_^2*α*^, and Δ for metamemory performance.

	Do not know		Do not know correct		TOT number		TOT strength		Gamma	
	PD	Elderly	PD	Elderly	PD	Elderly	PD	Elderly	PD	Elderly
*M*	.36	.39	.22	.23	.16	.12	12.00	13.37	.72	.75
SD	.22	.20	.12	.12	.11	.07	2.75	4.66	.44	.40
SE	.05	.04	.03	.03	.02	.01	0.61	1.04	.10	.10
*E* ^*α*^	.69		.69		.70		0.70		.69	
*E^2^* ^*α*^	.69		.69		.71		0.71		.69	
95% CI	.26–.46	.31–.48	.17–.28	.18–.29	.11–.21	.09–.15	10.72–13.28	11.19–15.55	.50–.93	.55–.95
95% ICI	.29–.43	.33 –.45	.18–.26	.19–.27	.13–.20	.09–.14	11.09–12.91	11.83–14.91	.57–.87	.61–.89
90% ICI	.31–.42	.34–.44	.19–.25	.20–.26	.13–.19	.10–.14	11.24–12.76	12.09–14.65	.60–.84	.63–.87
_*e*_ *R*_*g*_^2*α*^	.14		.07		.09		3.41		.27	
Δ	.17		.11		.06		4.36		.40	

Note: *N* = 22 except that for TOT strength *N* = 20 for the PD group and *N* = 20 for the elderly group and for gamma, *N* = 18 for the PD group and *N* = 17 for the elderly group; *E* ^*α*^ is based on 100  (1 − *α*) and *E* ^2*α*^ is based on (1 − 2*α*); _*e*_ *R*_*g*_^2*α*^ is based on 90% ICI and Δ is based on 95% CI for the elderly group.
